# Pilot Study: Algorithm-Based Assessment of Maxillary Sinus Volume in Zygomatic and Pterygoid Implant Planning

**DOI:** 10.3390/dj13110515

**Published:** 2025-11-05

**Authors:** Pablo García Roza, Iago Vila García, Miguel González Menéndez, Jesús Pato Mourelo, Jose Antonio Vega, M. Zulima Fernández-Muñiz

**Affiliations:** 1 Departamento de Morfología y Biología Celular, Universidad de Oviedo, 33006 Oviedo, Asturias, Spain; uo172532@uniovi.es (P.G.R.); javega@uniovi.es (J.A.V.); 2 Grupo de Problemas Inversos, Optimización y Aprendizaje Automático, Departamento de Matemáticas, Universidad de Oviedo, 33006 Oviedo, Asturias, Spain; uo265800@uniovi.es; 3 Centro Ondontológico MG, 33005 Oviedo, Asturias, Spain; miguel@centromiguelgonzalez.com; 4 Galimplant, 27600 Sarria, Lugo, Spain; jpatomourelo@hotmail.com; 5 Facultad de Ciencias de la Salud, Universidad Autónoma de Chile, Providencia, Santiago 7500912, Chile

**Keywords:** zygomatic implants, maxillary sinus, CBCT, volume measurement, image segmentation

## Abstract

**Background/Objetives:** Zygomatic implants are an effective solution for the prosthetic rehabilitation of atrophic maxillae, but their placement can alter maxillary sinus anatomy and influence surgical outcomes. This study presents a computational algorithm for automated segmentation and volumetric assessment of the maxillary sinus from cone-beam computed tomography (CBCT) images, offering a reproducible and clinically oriented tool. **Methods:** Six sinus samples from four patients undergoing pterygoid or zygomatic implant surgery were analyzed. The algorithm was designed to integrate image binarization, surface detection, and iterative reconstruction to delineate sinus boundaries and compute volumes with minimal operator dependence. **Results:** Postoperative analyses consistently revealed reductions in sinus volume, with relative changes ranging from 1.1% to 24.5%, validated by expert review. **Conclusions:** These results demonstrate the feasibility of algorithm-driven volumetric assessment as a non-invasive approach to support surgical planning and monitor anatomical changes. Although limited by the small sample size, this pilot study establishes a foundation for further research and highlights the clinical potential of computational methods to enhance precision and safety in zygomatic implantology.

## 1. Introduction

Zygomatic implants (ZIs) are long, screw-shaped dental implants specifically designed for the prosthetic rehabilitation of patients with extensive maxillary defects of various etiologies [1,2,3]. Their indications have expanded beyond oncologic and traumatic cases to include edentulous [4,5,6,7] and partially edentulous patients with severely atrophic maxillae [8,9,10,11]. Reported clinical outcomes are generally favorable, with success rates ranging from 82% to 100% [4] and survival rates between 97.86% and 100% [12,13]. Nevertheless, these procedures may involve short- and long-term complications [14,15].

Several surgical techniques have been developed for ZI placement [16,17], including anatomically guided [18], computer-assisted [19], and template-based approaches [20], all aiming to achieve accurate positioning. In most cases, ZI placement involves traversing the maxillary sinus (trans-sinus approach), which, although considered invasive, has relatively low postoperative complication rates: 2.4% [14], 3.9% [21], and 11.4% [22] for sinusitis. The extra-sinus approach has been proposed to reduce these risks [22]. However, even in the absence of overt clinical complications, ZI placement can alter the anatomy and dimensions of the maxillary sinus. Jin et al. [23] analyzed the effects of ZI placement using radiographic and clinical indicators, reporting Schneiderian membrane thickening, an increase in type IV sinus morphology (fully radiopaque), infundibulum obstruction, and a low incidence of osteitis.

A detailed understanding of the maxilla–zygomatic complex is therefore essential for dental surgeons and maxillofacial specialists when planning surgical interventions involving zygomatic implants [24]. The anatomical complexity of the maxillary sinus and its proximity to critical neurovascular structures significantly increase the risk of complications [25,26]. In this regard, Rosano et al. [27] provided a comprehensive anatomical analysis of the maxillary sinus using CT imaging, highlighting the importance of pre- and postoperative evaluation to minimize surgical risks and improve sinus augmentation procedures.

The maxillary sinus is a key anatomical structure within the maxilla, contributing to respiration and craniofacial biomechanics. Its volume is particularly relevant for zygomatic implants, which serve as an alternative to conventional dental implants in patients with severe maxillary bone resorption. Since these implants are anchored in the zygomatic bone while passing through or closely interacting with the maxillary sinus, accurate volume measurement is essential to minimize complications and optimize implant placement. In this context, automated algorithms provide a precise and efficient tool for maxillary sinus volume analysis, allowing more reliable preoperative evaluation and improved surgical outcomes. Variations in maxillary sinus volume have been associated with anatomical factors such as nasal septum deviation, emphasizing the need for accurate three-dimensional assessment in surgical planning [28]. Recent studies highlight the value of CBCT imaging for anatomical evaluation of the maxillary sinus and its relationship to adjacent structures when planning zygomatic or sinus-related procedures [29]. Furthermore, deep learning-based segmentation approaches, such as U-Net architectures, have demonstrated high accuracy in delineating sinus boundaries in CBCT data [30].

Despite the progress achieved by deep learning approaches for sinus segmentation [30,31,32], their dependence on large, annotated datasets and computational resources limits their clinical transferability. Moreover, their internal decision processes are often non-transparent to clinicians. The present study addresses this gap by developing a deterministic, rule-based segmentation algorithm that enables interpretable, reproducible, and dataset-independent volumetric assessment of the maxillary sinus. To our knowledge, no previous work has applied such a computationally lightweight algorithm to quantify sinus volume variations following zygomatic or pterygoid implant surgery.

Recent research has focused on integrating advanced imaging techniques with computational optimization methods to enhance surgical planning. Machine learning and automated segmentation have been proposed to refine preoperative evaluation and implant positioning [31]. A significant contribution in this field is the development of an algorithm for volume estimation from DICOM data obtained through cone-beam computed tomography (CBCT). Initially validated for dental applications such as pulp chamber volume measurement—demonstrating high accuracy compared to micro-CT [32]—this algorithm employs image segmentation and region-growing techniques. It has now been adapted for maxillary sinus analysis to assess bone availability and spatial constraints for ZI placement.

The proposed algorithm-driven approach was implemented for the preoperative planning of zygomatic implants. Its performance was compared with conventional methods to demonstrate the potential benefits of computational optimization in implantology, improving precision, safety, and clinical outcomes.

The main objective was to evaluate changes in maxillary sinus volume before and after implant surgery using a newly developed automated segmentation algorithm. The null hypothesis stated that no statistically significant differences would be observed between preoperative and postoperative volumes as measured by the proposed method.

## 2. Materials and Methods

### 2.1. Study Population and Inclusion Criteria

The study was conducted between January 2023 and March 2025 at Centro Odontológico MG (Oviedo, Spain), in collaboration with the Section of Anatomy and Human Embryology of the Department of Morphology and Cellular Biology and the Department of Mathematics, University of Oviedo. A total of 103 patients were initially retrieved from two private maxillofacial surgery centers (private centers of authors P.G.-R. and M.G.-M.). All patients provided signed written informed consent, and all clinical procedures were performed in accordance with Spanish law, the principles of the Declaration of Helsinki, and the Good Clinical Practice (GCP) Guidelines.

Only four patients met the inclusion criteria: availability of a preoperative CBCT scan; surgical treatment involving complete extraction of the remaining dentition or edentulous maxillae at baseline; placement of at least one pterygoid implant; availability of a postoperative CBCT scan obtained at least six months after implant placement; and complete visualization of the affected maxillary sinus in both preoperative and postoperative scans.

Of these included cases, two patients underwent bilateral maxillary surgery in a single procedure. One patient was treated only in the right sinus, while another underwent bilateral intervention, although one of the sinuses did not meet the inclusion criteria. Details of the included cases are summarized in Table 1.

### 2.2. Algorithm for Detection of the Maxillary Sinus

To detect the area occupied by the maxillary sinus, the algorithm was applied to six samples, each comprising two sets of images: one acquired before the intervention and another obtained 14 to 16 months postoperatively.

**Figure 1:**
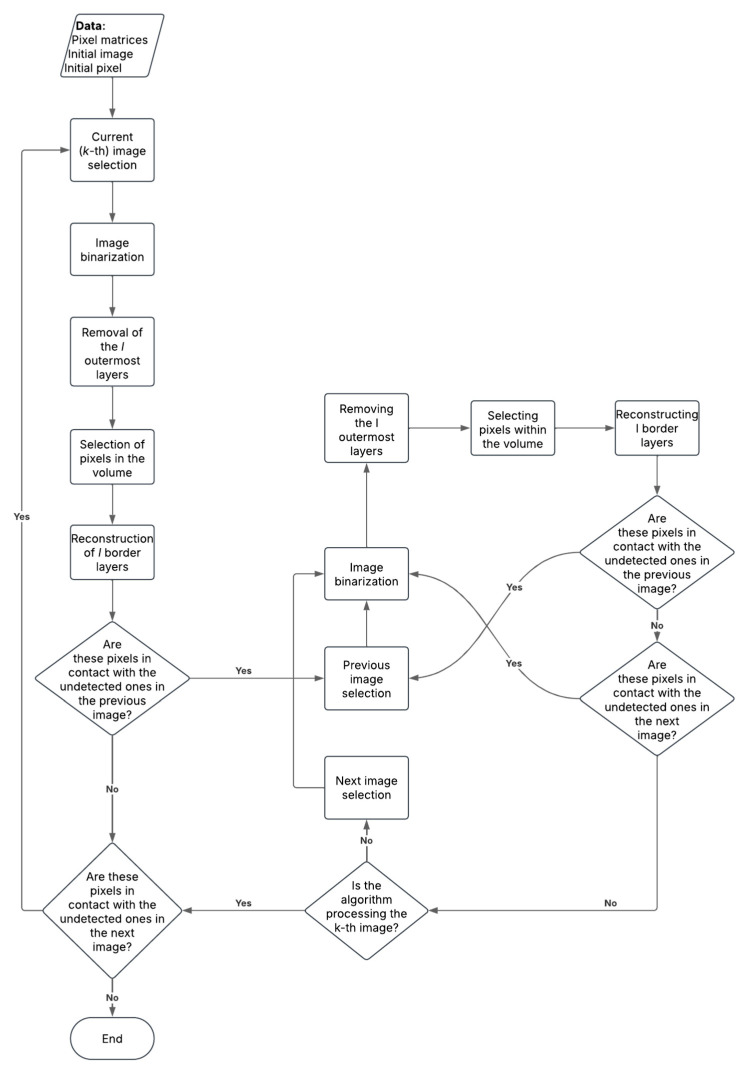
Flowchart of the surface detection algorithm used in this study.

The detection algorithm was designed to accurately identify the sinus boundaries and estimate its volume from medical imaging data. It follows a sequence of steps to ensure precise segmentation and reproducible volume calculation (see the algorithm flowchart in Figure 1).

1. **Input data**:

Image set: All images are grayscale, represented by matrices of integers ranging from 0 to 255, with each element corresponding to a pixel intensity.

Starting pixel and image: A pixel known to belong to the sinus must be selected from any image in the set to initiate volume detection.

Control parameter k: This user-defined threshold determines the binarization of the images, adjusted to match the segmented region to the target volume.

2. **Image binarization**: Each image matrix I is converted to a binary matrix M such that:

$$M_{ij}=\left\{\begin{array}{cc} 1 & \;\mathrm{if}\;I_{ij}\geq k,\\ 0 & \;\mathrm{if}\;I_{ij}\geq k \end{array}\right.$$

where k is manually selected.

3. Surface separation: For any nonzero element $a_{12}\in M$ with coordinates ($a_{1}{,a}_{2}$), the equivalence class defining a surface is as follows:

$$\left[a\right]=\left\{M_{ij}\in M\;|\;M_{ij}\neq 0,\exists a_{1},\ldots ,a_{n}\in M\;with\;a_{k}\neq 0\;\forall k\;\;=1,\ldots ,n\;verifying\;d_{1}(a_{1}{,a}_{2})\leq 1,\ldots ,d_{1}(a_{n}{,M}_{ij})\leq 1\right\}$$

where $d_{1}$ is the metric induced by the $L_{1}$ norm. The number of equivalence classes equals the number of nonzero elements in M, and each defines a surface.

To find all points in the same equivalence class as $M_{ij}$, the algorithm iteratively adds neighboring nonzero pixels (with $d_{1}=1$) to the class and sets them to zero in M to avoid repetition, continuing until no nonzero elements remain.

4. **Maxillary sinus identification in each image**: For a given image, surfaces associated with the sinus volume V are determined by checking whether pixels in the upper and lower planes are contiguous with pixels of equivalence classes in the current plane. Let $S={\left\{{\left[S\right]}_{j}^{k}\right\}}_{j=1}^{m}$ be the set of equivalence classes in plane k; then

$${\left[S\right]}^{k\pm 1}\subset V↔\exists a\in {\left[S\right]}^{k\pm 1},b\in {\left[S\right]}_{j}^{k}\;j=1,\ldots m\;|\;d_{1}(a,b)\leq 1$$

In the first iteration, only the surface containing the initial pixel is retained; subsequent iterations follow the same approach.

To automatically remove connections to other cavities separated by invisible septa, the following procedure is applied:

Starting from the binarized matrix $M^{k,0}$, a new matrix $M^{k,l}$ is constructed:

$$M_{ij}^{k,l}=\left\{\begin{array}{cc} 1 & \;\mathrm{if}\;M_{i-1,j}^{k,l-1}+M_{i+1,j}^{k,l-1}+M_{i,j-1}^{k,l-1}+M_{i,j+1}^{k,l-1}=4\\ 0 & \;\mathrm{otherwise} \end{array}\right.$$

**Figure 2:**
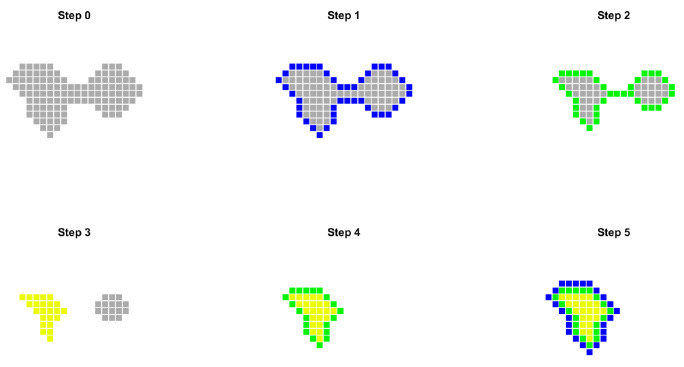
Example of the layer removal–reconstruction procedure. Step 0 shows the detection of two surfaces connected by a channel that does not belong to the segmented region. Steps 1 and 2 illustrate the removal of the two outermost layers. Step 3 shows the selection of the surface of interest. Steps 4 and 5 correspond to the layer reconstruction process. After completion, the connection between the two surfaces has been removed.

This removes the l outermost layers of each surface (Steps 1–2 in Figure 2).

Using the adjacency criterion, a matrix ${\overset{ˇ}{M}}^{k,l}$ is generated, containing only the sinus pixels (Step 3).

Layer reconstruction is performed with reference to $M^{k,0}$. For each nonzero element $a_{ij}^{k,l}\in {\overset{ˇ}{M}}^{k,l}$ satisfying

$$a_{i-1,j}^{k,l}+a_{i+1,j}^{k,l}+a_{i,j-1}^{k,l}+a_{i,j+1}^{k,l}<4$$

all neighboring nonzero pixels b in the original matrix $M^{k,0}$ with $d_{1}\left(a_{ij}^{k,l},b\right)=1$. This repeats iteratively until reaching ${\overset{ˇ}{M}}^{k,0}$, restoring all removed layers (Steps 4–5). This approach ensures that only the true sinus is retained while automatically eliminating unwanted connections. Evaluation of upper and lower plane pixels is restricted to a window around potential connections, not the entire image, improving accuracy.

5. **Iterative volume refinement**: After each iteration, newly detected pixels are checked for contact with previously undetected equivalence classes in adjacent slices. If such contact exists, the algorithm traverses the images in reverse to incorporate them. This process continues until no new pixels are added. Finally, the images are scanned in normal order to associate any remaining pixels with the sinus volume.

Depending on the nature of the images being processed, it may be advisable to perform manual corrections in those cases where the algorithm cannot achieve complete segmentation accuracy automatically. Once the images resulting from the detection step have been obtained, the volume is calculated as described below.

Before computing the volumes, segmentation accuracy was independently assessed by two expert radiologists, who manually delineated the maxillary sinus boundaries on the same CBCT slices. Inter-observer agreement was quantified using Cohen’s kappa coefficient:

$$\kappa =\frac{p_{0}-p_{e}}{1-p_{e}}$$

where $p_{0}$ is the proportion of observed agreements between the experts (i.e., the number of voxels where both raters assigned the same label divided by the total number of voxels), and $p_{e}$ is the proportion of agreements expected by chance if both raters were segmented randomly. A kappa value of κ = 0.943 ± 0.104 indicated substantial consistency between the experts [33]. A consensus segmentation, defined as the union of both manual annotations, was subsequently used as the reference standard.

The Dice Similarity Coefficient (DSC) was then computed for each case as

$$DSC=\frac{2\left|A\cap B\right|}{\left|A\right|+\left|B\right|}$$

where A denotes the set of voxels from the automated segmentation and B the set of voxels from the consensus expert segmentation. The resulting mean DSC of 0.971 ± 0.020 demonstrated a high degree of overlap between automated and expert segmentations, confirming the reliability and reproducibility of the proposed method.

The algorithm was implemented in MATLAB R2023b (MathWorks, Natick, MA, USA) using custom scripts and MATLAB’s Image Processing Toolbox for matrix-based operations, binarization, and morphological analysis. All computational parameters, environment details, and validation metrics (Cohen’s κ and DSC) are provided to ensure full reproducibility.

### 2.3. Algorithm for Volume Calculation

The following volume computation was performed on the segmentations previously validated by the two maxillofacial surgery experts, ensuring that only anatomically consistent sinus boundaries were included in the analysis.

To calculate the volume, the analysis is performed on the segmentation obtained from the maxillary sinus images. Under the assumption that detection errors occur only at the boundary pixels, the true volume is considered to lie at the midpoint of an interval defined by two extreme volumes.

Since potential errors are located at the boundary, some pixels included in the detection may not actually belong to the object whose volume is being measured. Accordingly, the lower bound of the volume is defined by removing all boundary pixels from the original segmentation. Specifically, given the detection matrix ($M_{ij}$), a new matrix ($M_{ij}^{lower}$) is constructed as follows:

$$M_{ij}^{lower}=\left\{\begin{array}{cc} 1 & \;\mathrm{if}\;{M_{ij}+M}_{i-1,j}+M_{i+1,j}+M_{i,j-1}+M_{i,j+1}=5\\ 0 & \;\mathrm{otherwise} \end{array}\right.$$

This operation removes the outermost layer of the detected region. Conversely, the upper bound of the volume is obtained by adding non-detected pixels contiguous to the boundary of the original segmentation; that is, given $M_{ij}$, the matrix $M_{ij}^{upper}$ is constructed as follows:

$$M_{ij}^{upper}=\left\{\begin{array}{cc} 1 & \;\mathrm{if}\;{M_{ij}+M}_{i-1,j}+M_{i+1,j}+M_{i,j-1}+M_{i,j+1}\geq 1\\ 0 & \;\mathrm{otherwise} \end{array}\right.$$

This extends the detected region by one additional layer.

Finally, the volume is calculated as the mean of the upper and lower bounds, with the associated error estimated as half the width of this interval. Each detected pixel is assumed to contribute a cubic volume with side length L, and the total volume is obtained by summing over all detected pixels.

## 3. Results

**Figure 3:**
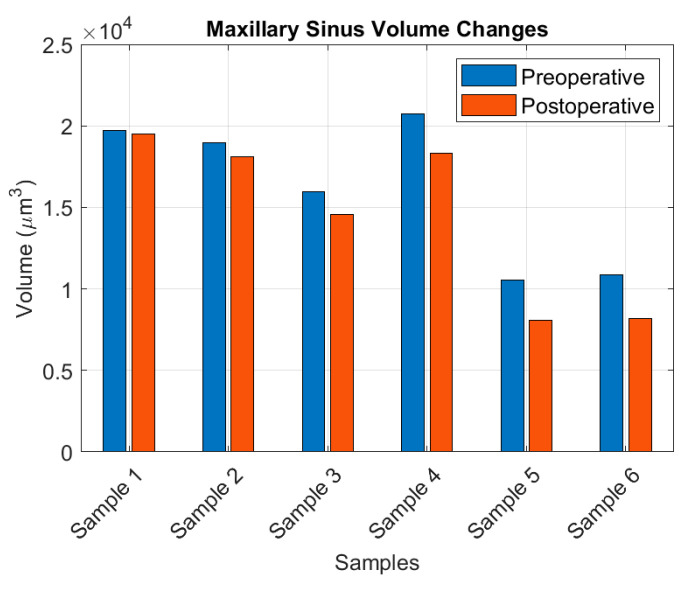
Postoperative changes in maxillary sinus volume represented in bar chart form. This graphical depiction complements Table 2 and enhances the readability and interpretation of the results.

Table 2 summarizes the maxillary sinus volumes calculated for the six samples corresponding to the four patients included in the study, both prior to surgery and after a follow-up period of approximately 14–16 months. To complement the tabular data, Figure 3 has been added to present the postoperative sinus volume changes in graphical form (bar chart), enhancing the readability and interpretation of the results.

The results indicated a consistent reduction in maxillary sinus volume across all patients. The relative decrease is expressed as the change with respect to the original sinus volume. The observed variation exhibits notable dispersion, which can be attributed to several factors: intrinsic inter-patient anatomical differences, heterogeneity in bone regeneration and remodeling, variability in surgical technique, and differences in postoperative healing dynamics.

**Figure 4:**
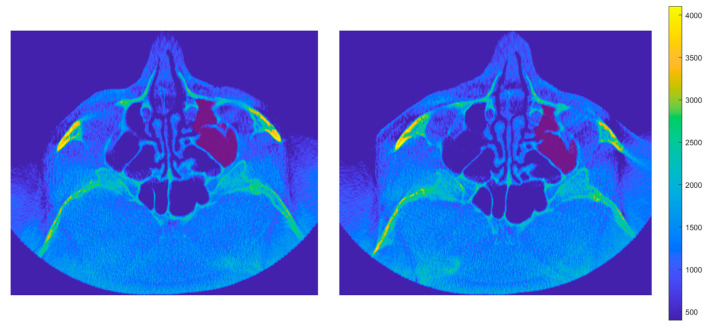
Coronal CBCT sections of the same patient before (**left**) and after (**right**) zygomatic implant placement. The central reference plane (0) defines the orientation of the image stack, from which more caudal ($0-x_{d}$) and cranial ($0+x_{u}$) sections are obtained. The color representation varies according to visualization mode. In standard radiological grayscale, lighter tones correspond to denser, more radiopaque tissues (bone and calcifications), while darker tones represent less dense soft tissues. In MATLAB-based visualization, a colormap from blue (low density) to yellow (high density) is applied, with red-shaded regions highlighting the air-filled maxillary sinus cavity. For expert evaluation, this color scale is simplified to black and dark blue hues, preserving red as the marker of the sinus cavity analyzed in this study.

To illustrate the anatomical reference used for segmentation, Figure 4 shows two adjacent coronal CBCT planes of the same patient before and after zygomatic implant placement. A central reference plane (image 0) defines the orientation of the image stack, from which more caudal ($0-x_{d}$) and cranial ($0+x_{u}$) sections are obtained. This representation clarifies how the algorithm interprets consecutive planes from inferior to superior levels when identifying the maxillary sinus boundaries.

**Figure 5:**
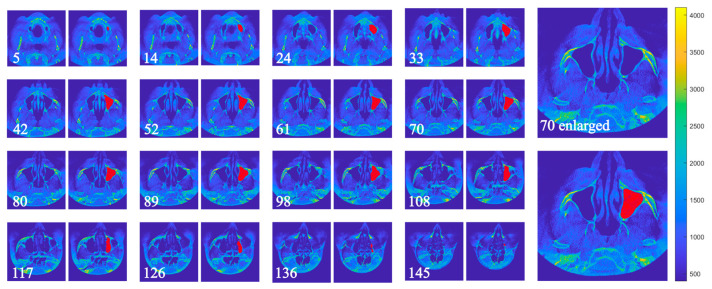
Preoperative maxillary sinus of Sample 3, with detection results using the described algorithm. For consistency, the slice orientation has been standardized to the coronal plane.

**Figure 6:**
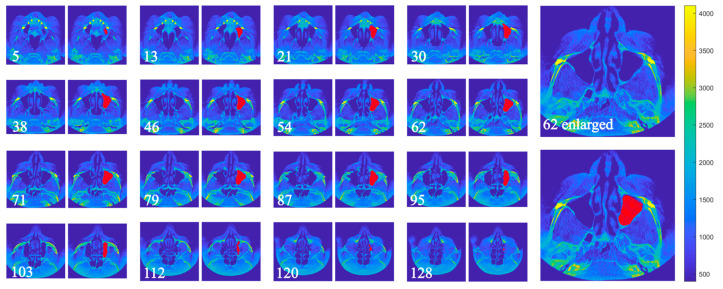
Postoperative maxillary sinus of Sample 3, 16 months after surgery, with detection results using the described algorithm. For consistency, the slice orientation has been standardized to the coronal plane.

Figure 5 and Figure 6 illustrate the segmentation of the maxillary sinus before and after surgery using the proposed algorithm. The segmented area was closely aligned with the actual sinus boundaries, and segmentation accuracy was validated by an expert. The examples correspond to Patient 2, Sample 3, left side. Non-consecutive slices were intentionally selected to provide a clearer visualization of the algorithm’s processing steps and the resulting segmentation. Additionally, one image on the right side is displayed at a larger scale to highlight the segmented region in greater detail.

These results demonstrated that the algorithm effectively captures volumetric changes in the maxillary sinus over time, supporting its potential utility for preoperative planning and postoperative assessment in zygomatic implantology. The method allows for quantitative evaluation of anatomical modifications that may impact implant placement and long-term outcomes.

## 4. Discussion

In the present study, the objectives were twofold: first, to introduce an efficient, cost-effective, and straightforward algorithm for quantifying sinus cavity volumes from CBCT images; and second, to evaluate the biological changes occurring in the maxillary sinuses following pterygoid implant surgery. Analysis of pre- and postoperative sinus volumes in the six included samples revealed a general trend toward volume reduction, although substantial inter-individual variability was observed. The relative change in volume, summarized in Table 2 and computed as

$$∆V_{rel}=\frac{V_{pre}-V_{post}}{V_{pre}}$$

where $V_{pre}$ and $V_{post}$ denote the preoperative and postoperative volumes, respectively, showed an average reduction of 12.4% ($∆V_{rel}$ = 0.124), with a standard deviation of 9.6 percentage points ($s_{∆V}$ = 0.096). The 95% confidence interval, estimated using the Student’s t-distribution with df = 5,

$${CI}_{95\%}=∆{\bar{V}}_{rel}\pm t_{0.0025,5}\cdot \frac{s_{∆V}}{\sqrt{n}}=0.124\pm 0.101$$

ranged from 2.3% to 22.5%, reflecting both biological variability and the limitations of a small cohort. Some patients exhibited minimal change, whereas others experienced reductions exceeding 24%, likely due to anatomical differences or individual biological factors.

As this study included only six patients, its findings should be interpreted with caution. It was intentionally designed as a pilot investigation aimed at demonstrating methodological feasibility and identifying general trends rather than drawing statistically generalizable conclusions. Nevertheless, even within this limited cohort, a consistent tendency toward postoperative reduction in maxillary sinus volume could be observed, supporting the potential biological relevance of the proposed quantitative approach.

Descriptive statistics further indicated that the mean preoperative volume was (${\bar{V}}_{pre}$ = 16,137.7 µm^3^) (SD: 4511.7 µm^3^), decreasing postoperatively to ${\bar{V}}_{pre}=$ 14,459.3 µm^3^) (SD: 5167.6 µm^3^). While the small sample size limits generalizability, the combined use of mean values, standard deviations, and confidence intervals provides a coherent quantitative picture, supporting the conclusion that the intervention tends to reduce sinus volume on average.

Maxillary sinus measurements using CBCT have been shown to be clinically valid, providing higher resolution than standard multidetector CT [34,35,36,37]. Dr. Lucía Hernández [38] demonstrated that CBCT-based measurements of pulp chambers closely resemble micro-CT reference values, with relative errors below 5.7%, supporting the reliability of CBCT for volumetric assessment. Nevertheless, unclear anatomical boundaries and the presence of high-contrast elements, such as intraoral implants, may introduce operator-dependent variability. Limitations of the study include the small sample size and the occasional need for expert intervention in cases with ambiguous anatomical boundaries or high-contrast artifacts.

Recent studies have highlighted the effectiveness of deep learning architectures, such as U-Net and its variants, for medical image segmentation, achieving high accuracy in large annotated datasets [30,31]. However, these approaches typically require extensive manual labeling, GPU-based computational resources, and large training cohorts to generalize across patients and imaging conditions. In contrast, the deterministic, rule-based algorithm presented here does not rely on prior training or large datasets, making it particularly suitable for pilot studies and small-sample clinical scenarios. Its fully interpretable and reproducible workflow enables transparent validation of each segmentation step—an advantage not always present in deep learning approaches.

Moreover, previous U-Net-based segmentation approaches for the maxillary sinus have reported Dice coefficients in the range of 0.96–0.99 on large annotated datasets [30,39]. In the present study, the deterministic algorithm achieved a comparable mean Dice Similarity Coefficient of 0.97 ± 0.02, despite requiring no training data. This indicates that rule-based segmentation can yield competitive accuracy while offering advantages in interpretability, reproducibility, and computational simplicity.

Future research should aim to integrate this deterministic algorithm as a preprocessing or refinement stage within hybrid deep learning pipelines, combining the explainability and reproducibility of rule-based methods with the adaptive feature extraction capabilities of convolutional neural networks. Moreover, larger cohorts would allow more robust statistical analyses, facilitate the identification of factors underlying variability, and potentially enable predictive modeling for personalized clinical decision-making.

Overall, these findings indicate that the proposed algorithm is an efficient and potentially valid method for assessing maxillary sinus volume changes following surgery, while also providing a framework for integrating deterministic and learning-based approaches in future studies.

## 5. Conclusions

This study introduces an automated CBCT-based algorithm for the detection and volumetric quantification of the maxillary sinus in patients undergoing zygomatic and pterygoid implant surgery. The method combines image binarization, surface separation, and layer reconstruction to achieve accurate, reproducible, and minimally operator-dependent segmentation.

Analysis of six sinus samples revealed a consistent postoperative reduction in maxillary sinus volume, with relative changes ranging from 1.1% to 24.5%. These results suggest that implant placement may induce measurable anatomical modifications within the sinus cavity, highlighting the importance of precise preoperative evaluation.

Despite the small sample size and the occasional need for expert intervention in cases with unclear boundaries or high-contrast artifacts, the findings demonstrate the feasibility of algorithm-driven volumetric assessment. This computational approach provides a reliable, non-invasive tool that can enhance surgical planning and postoperative follow-up. Future research involving larger cohorts and the integration of machine learning techniques could potentially further validate, extend, and strengthen its clinical applicability.

## Figures and Tables

**Table 1:** Analyzed cases included according to year of birth, sex, and operated jaw.

Patient ID	Sample ID	Year of Birth	Sex	Operated Jaw
Patient 1	Sample 1	1950	Female	Left
Patient 1	Sample 2	1950	Female	Right
Patient 2	Sample 3	1952	Male	Left
Patient 2	Sample 4	1952	Male	Right
Patient 3	Sample 5	1966	Male	Right
Patient 4	Sample 6	1947	Female	Right

**Table 2:** Maxillary sinus volumes before surgery and 14–16 months postoperatively, with absolute values and relative changes. Volumes are expressed in µm^3^.

Sample	Preoperative Volume (µm^3^)	Postoperative Volume (µm^3^)	Relative Change (%)
Sample 1	19,734.1 ± 1094.5	19,521.9 ± 1126.5	1.1
Sample 2	18,990.2 ± 1004.6	18,087.4 ± 1006.8	4.8
Sample 3	15,959.7 ± 933.9	14,552.6 ± 899.6	8.8
Sample 4	20,756.2 ± 1111.5	18,313.2 ± 1098.9	11.8
Sample 5	10,525.6 ± 420.2	8,083.3 ± 510.9	23.2
Sample 6	10,860.4 ± 750.4	8,197.4 ± 430.2	24.5

## Data Availability

The original contributions presented in this study are included in the article. Further inquiries can be directed to the corresponding author.
